# Cerebral Complications of Snakebite Envenoming: Case Studies

**DOI:** 10.3390/toxins14070436

**Published:** 2022-06-27

**Authors:** Yu-Kai Huang, Yen-Chia Chen, Chia-Chun Liu, Hui-Chun Cheng, Anthony T. Tu, Kun-Che Chang

**Affiliations:** 1 Graduate Institute of Medicine, College of Medicine, Kaohsiung Medical University, Kaohsiung 80708, Taiwan; yukaih@gmail.com; 2 Division of Neurosurgery, Department of Surgery, Kaohsiung Medical University Hospital, Kaohsiung 80708, Taiwan; 3 Department of Surgery, Kaohsiung Municipal Ta-Tung Hospital, Kaohsiung 80145, Taiwan; 4 Emergency Department, Taipei Veterans General Hospital, Taipei 11217, Taiwan; ycchen4@gmail.com; 5 Department of Emergency Medicine, School of Medicine, National Yang-Ming University, Taipei 11221, Taiwan; 6 National Defense Medical Center, Taipei 11490, Taiwan; 7 Department of Ophthalmology, Louis J. Fox Center for Vision Restoration, University of Pittsburgh School of Medicine, Pittsburgh, PA 15213, USA; chl373@pitt.edu (C.-C.L.); huc58@pitt.edu (H.-C.C.); 8 Department of Biochemistry and Molecular Biology, Colorado State University, Ft. Collins, CO 80523, USA; atucsu@gmail.com; 9 Department of Neurobiology, Center of Neuroscience, University of Pittsburgh School of Medicine, Pittsburgh, PA 15213, USA

**Keywords:** snakebite envenoming, hemotoxin, neurotoxin, ischemic stroke, intracranial hemorrhage, anti-venom serum

## Abstract

There are an estimated 5.4 million snakebite cases every year. People with snakebite envenoming suffer from severe complications, or even death. Although some review articles cover several topics of snakebite envenoming, a review of the cases regarding cerebral complications, especially rare syndromes, is lacking. Here, we overview 35 cases of snakebite by front-fanged snakes, including *Bothrops*, *Daboia*, *Cerastes*, *Deinagkistrodon*, *Trimeresurus*, and *Crotalus* in the *Viperidae* family; *Bungarus* and *Naja* in the *Elapidae* family, and *Homoroselaps* (rare cases) in the *Lamprophiidae* family. We also review three rare cases of snakebite by rear-fanged snakes, including *Oxybelis* and *Leptodeira* in the *Colubridae* family. In the cases of viper bites, most patients (17/24) were diagnosed with ischemic stroke and intracranial hemorrhage, leading to six deaths. We then discuss the potential underlying molecular mechanisms that cause these complications. In cases of elapid bites, neural, cardiac, and ophthalmic disorders are the main complications. Due to the small amount of venom injection and the inability to deep bite, all the rear-fanged snakebites did not develop any severe complications. To date, antivenom (AV) is the most effective therapy for snakebite envenoming. In the six cases of viper and elapid bites that did not receive AV, three cases (two by viper and one by elapid) resulted in death. This indicates that AV treatment is the key to survival after a venomous snakebite. Lastly, we also discuss several studies of therapeutic agents against snakebite-envenoming-induced complications, which could be potential adjuvants along with AV treatment. This article organizes the diagnosis of hemotoxic and neurotoxic envenoming, which may help ER doctors determine the treatment for unidentified snakebite.

## 1. Introduction

Snakes are limbless reptiles that can be found on all continents and oceans except Antarctica. Among the 3700 species of snakes, 15% of them are estimated to be venomous [1]. On the continents, *Viperidae* and *Elapidae* are the major venomous snake families in the world [2]. Several hundred thousand people suffer from snakebite envenoming every year, and 5% of victims die due to the accompanying complications [3,4]. Among the five continents, southern Asia unfortunately reports the most deaths from snakebites [3]. However, with the invention of antivenom (AV), survival rates following a venomous snakebite have significantly increased [5].

Snake venoms are complex mixtures of enzymes, lipids, nucleotides, and carbohydrates. There are three main toxins in snake venom, including hemotoxins, neurotoxins, and cytotoxins [6], which lead to systematic damage, including cerebral complications. Among the complications, cerebral hemorrhage, ischemic stroke, cerebral infarction, and secondary inflammation frequently occur after viper envenomings due to hemotoxic enzymes such as snake-venom metalloproteinases (SVMPs) [7], coagulant enzymes [8], and proteolytic enzyme toxicity [9]. Without appropriate treatment, these compounds cause severe irreversible brain edema and fatality [10,11,12,13,14].

Some venomous snake bites treated with AV still result in fatality. Therefore, there remains an unmet need to develop an adjuvant therapy for snakebite envenoming. Most viper-bite cases studied, in both clinical reports and animal studies, were reported and diagnosed with hemorrhage and tissue necrosis in skin and muscle [15,16,17]. Little is known about viper-bite-induced complications in the central nervous system (CNS). In this article, we review a series of clinical snakebite cases that were reported after 2000, focusing on the cerebral complications caused by hemotoxic and neurotoxic envenoming, and describe a few cases of CNS and PNS complications of snakebite envenoming. Finally, we discuss other potential therapeutic strategies for snakebite treatment.

## 2. Envenoming by Snakes That Mainly Have Hemotoxic Venoms

Here, we review snakebites by three genera of the *Viperidae* family (Table 1).

### 2.1. Bothrops

*Bothrops*, commonly known as pit viper, *fer-de-lance* “spearhead” in French, is often found in central and south America. In Brazil, around 26,000 snakebites by *Bothrops jararaca* are recorded each year [22]. Due to the snake-venom metalloproteinases (SVMPs) and hemotoxins in venom, intracranial hemorrhage and infarction are the main brain complications after snakebite. The fatality rate of *Bothrops* bites is estimated to be about 0.51% [23]. Here we reviewed three cases of *Bothrops* bites that happened in Brazil and Martinique.

### 2.2. Deinagkistrodon

*Deinagkistrodon acutus* is a *Viperidae* family snake endemic to Southeast Asia. It is also called the “hundred pacer” because locals believe that the victims who are bitten will only be able to walk 100 steps before dying. Although *D. acutus* presents hemotoxins in the venom [24], fatality is unusual after *D. acutus* bite. Here, we review one case of *D. acutus* bite that happened in China.

### 2.3. Trimeresurus

*Trimeresurus stejnegeri*, also called bamboo viper, is endemic to southern China and Taiwan. Due to the similar color of *T. stejnegeri* to bamboo, victims are typically bitten while walking in a bamboo forest. SVMPs and hemotoxins are the main toxins in the *T. stejnegeri* venom. A bite wound usually becomes swollen and gradually undergoes necrosis [25]. The severity of the wound depends on the amount of venom injected and the depth of the bite. Here, we review one case of *T. stejnegeri* bite that happened in China.

## 3. Envenoming by Snakes That Mainly Have Neurotoxic Venoms

Here, we review snakebites by two different genera of the *Elapidae* family (Table 2).

### 3.1. Bungarus

Three cases were reported in Thailand that involved *Bungarus Candidus* [26], which is also called Malaya Krait and is often found in Southeast Asia. Two patients were retreated with AV but still suffered from respiratory paralysis and ptosis and were discharged with permanent mydriasis. Another patient did not receive AV and was diagnosed with prolonged cerebral anoxia. Unfortunately, she was discharged with permanent brain damage. Other symptoms such as tachycardia and periodic convulsions were also noted in the report. Neurotoxins are enriched in Elapid snakes’ venom [32]. Phospholipase A2 (PLA_2_) is one of the neurotoxins found in the venom of *B. Candidus*, which causes neural disorders [33]. A previous review article also pointed out that PLA_2_ may cause ophthalmic disorders [34], which explains why the patients were discharged with permanent mydriasis.

In India, four patients were bitten by *B. caeruleus* (Indian Krait), one of the most common snakes in Bangladesh and India. A 15-year-old boy was bitten by *B. caeruleus* and immediately sent to the hospital to receive AV treatment [28]. After one vial of AV treatment, he had respiratory difficulty, perhaps due to a hypersensitivity reaction. He was diagnosed with hypoxia ischemic encephalopathy along with paraplegia. He was then discharged with vision loss in both eyes. Another 10-year-old boy was bitten by *B. caeruleus* on his ear while sleeping and sent to the hospital to receive a total of 10 vials of AV [29]. He was then diagnosed with PRES, a rare syndrome associated with Elapid snakebites. He recovered and was discharged 10 days later with minor vision blurring. Another two adult cases of *B. caeruleus* bite were treated with AV and diagnosed with early-morning neuroparalytic syndrome (EMNS) [30]. Although having difficulty in swallowing, double vision, and mydriasis, they were discharged with a full recovery.

In Vietnam, a 17-year-old girl was bitten by *B. multicinctus* (Many-banded Krait) and sent to the hospital a few hours later [27]. She did not receive AV treatment due to an unavailability at that time in Vietnam. She was stable for the first 2 days and suddenly deteriorated with seizures and a coma. She was then diagnosed with hyponatremia and cerebral edema. Unfortunately, she still did not make it 18 days later due to lack of AV treatment.

### 3.2. Naja

*Naja*, also known as cobra, is one of the most common elapid snakes on the planet. To date, there are about 38 species of *Naja* [35]. Here, we review a case of a 57-year-old woman that was bitten by *Naja arabica* (Arabian cobra) [31]. She was sent to the hospital one hour after *N. arabica* bite and immediately received multiple vials of AV treatment. After 6 h of intensive care (30 vials of AV), she was still diagnosed with brain death along with respiratory failure. She then received extensive AV treatment; up to 50 vials in 24 h. Fortunately, her neurologic status gradually recovered by the second day and her respiratory muscles started to function by the third day. She was then discharged from the ICU and eventually returned home with a full recovery.

## 4. Envenoming by Snakes That Have Both Hemotoxic and Neurotoxic Venoms

Here we review snakebites by 3 genera of the *Viperidae* family and 1 genus of the *Lamprophiidae* family (Table 3).

### 4.1. Daboia

*Daboia russelii*, also called Russell’s viper, is one of the most common vipers found in India [49] and is the greatest contributor to accidental snakebites [50]. To shorten the time needed to treat an unidentified snakebite, a group in Taiwan utilized avian and mammal antibodies to develop a rapid and sensitive diagnostic tool for a Russell’s viper’s snakebite [51]. Although *D. russelii* belongs to the family of *Viperidae*, several neurotoxins are identified in the viper’s venom [52] which also cause neural disorders such as ptosis and ophthalmoplegia [12,38]. Here, we review nine cases of *D. russelii* snakebites in India and one case in Sri Lanka. Since *D. russelii* is usually found on farms, 8 of the 10 patients were male farmers, and all were bitten on their feet. Because AV against *D. russelii* is well-established, most of the patients received AV during hospitalization and recovered with no or minor complications.

### 4.2. Cerastes

*Cerastes cerastes*, also called the desert horned viper due to its appearance, is endemic in the deserts of northern Africa and parts of the Arabian Peninsula. *Cerastes* is considered a venomous snake due to its hemotoxin venoms. In addition to SVMPs and hemotoxin, phospholipase A2 (PLA_2_) was found to be the main constituent of the venom [53]. Here we reviewed four patients who suffered from *Cerastes* bites. Receiving no AV treatment, two patients died after alternative medical treatments, and one recovered without any neurological complications. The patient who received AV and other medications was discharged with visual impairment, which might have been caused by PLA_2_ toxicity [34].

### 4.3. Crotalus

*Crotalus adamanteus*, also called Eastern diamondback rattlesnake, is endemic to the southeastern United States. Although AV against *C. adamanteus* is available, there are still fatalities due to snakebite-envenoming-induced intracranial hemorrhage [54]. Most venomous snakebite victims in the United States are bitten by a member of the rattlesnake family. However, with the development of AV, death from a *C. adamanteus* bite is rare [55]. Here we review one case of *C. adamanteus* bite that happened in the state of Florida. In addition to hemotoxins, neurotoxins are also the main components in the venom of *Crotalus*. Without severe cerebral complications, rattlesnake bites cause myokymia [45], fasciotomy-requiring compartment syndrome [46] and bilateral ptosis [47]. Even without AV treatment, they were all recovered.

### 4.4. Homoroselaps

*Homoroselaps*, belonging to the *Lamprophiidae* family, is mainly endemic to the Republic of South Africa. Despite having front fangs [56], *Homoroselaps* bite is unlikely to cause life-threatening effects due to the tiny amount of venom injected. Two adult males were bitten by *Homoroselaps lacteus* on their hands in South Africa [48]. One had numbness on his finger but recovered soon after. However, another patient had severe subcutaneous ecchymoses for 4 days. The ecchymoses were visible for two weeks on his hand before he recovered. Although the composition of the venom in *H. lacteus* has yet to be studied, SVMP and PLA_2_ were predicted in the venom due to the symptoms caused by the snakebite [48]. Other old studies of *H. lacteus* bite also reported bruises and/or ecchymoses on patients’ hands, which confirm the predictive components of the venom.

## 5. Envenoming by Rear-Fanged Snakes

Here we review three cases of rear-fanged snakebites by two genera of the *Colubridae* family (Table 4).

### 5.1. Oxybelis

*Oxybelis*, commonly known as the vine snake, is a genus of colubrid snakes mainly endemic to the Americas. Here, we review a case of 67-year-old male that was bitten on his arm by *Oxybelis fulgidus* in Brazil [57]. He came to the local hospital with bite-site bleeding and dizziness. Only taking painkiller pills, he recovered and was discharged in 2 days. No neurologic complication was observed during his hospitalization. Both hemotoxic and neurotoxic proteins such as L-amino acid oxidase (LAAO), PIII-SVMP, cysteine-Rich Secretory Proteins (CRiSP), and three-finger toxins (3FTx, fulgimotoxin) were identified in the venom of *O. fulgidus* [60,61].

### 5.2. Leptodeira

*Leptodeira*, commonly called the cat-eye snake, is a genus of colubrid snakes mainly endemic to Mexico and Central America. Here, we report two cases of *Leptodeira* bite, one by *Leptodeira annulate* and one by unknown species. A 29-year-old female was bitten on her finger by *L. annulate* while trying to catch the snake during fieldwork in Colombia [58]. She had edema on her whole hand 18 min after the bite and was treated with a single intravenous dose of hydrocortisone. She recovered and was discharged after 12 h and no systemic symptoms were referred. Another case was a 44-year-old male that was bitten by unidentified *Leptodeira* on his arm [59]. He had fever, chills, nausea, and light-headedness. He was then discharged soon later. However, a few months later, he complained about hyperalgesia, swelling, alternating cool and warm sensation, and dermal changes, and was then diagnosed with complex regional pain syndrome (CRPS), which is a rare case reported by snakebites in the world [59]. In addition, two cases of pediatric CRPS (<18-year-old) were reported to be bitten by a viper and southern Pacific rattlesnake in Turkey [62] and the US [63], respectively.

### 5.3. Other Studies of Colubridae Family

Compared with the venoms of many front-fanged snakes, the toxic nature of the venom in rear-fanged colubrid snakes is less clearly understood. It may be because of the difficulty of collecting the venom from rear-fanged snakes and of the tiny amount of venom for each injection. Like *Viperiade* snakes, SVMP is one of the main toxins in the venom, which was discovered in several colubrid species including *Dispholidus typus* [64], *Thamnodynastes strigatus* [65] and *Philodryas* species [66]. In addition to SVMP, other toxins such as serine proteases, LAAOs, PLA_2_, C-type lectin-like proteins and 3FTx were identified in colubrid snakes [60,61,67]. Although no clinical case of cerebral complications was reported by colubrid bite due to a tiny amount of envenoming, an animal model of venom (*Philodryas patagoniensis*) injection demonstrates the hemotoxic features of the venom, leading to multiple hemorrhages in the cerebellum and cerebrum [68].

## 6. Brain Complications

We review 35 cases of brain complications caused by toxic snake proteins, including hemotoxins (Table 1), neurotoxins (Table 2), and both toxins (Table 3 and Table 4).

### 6.1. Cerebral Infarction and Ischemic Stroke

**Figure 1:**
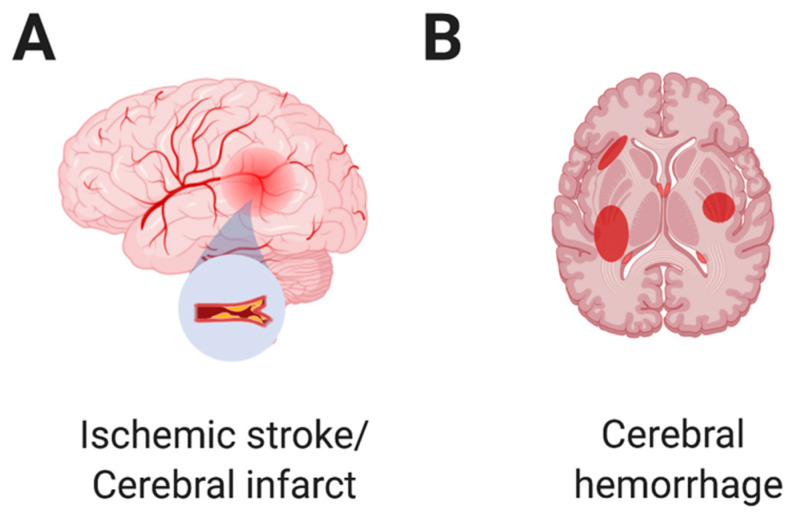
Main cerebral complications of snakebite envenoming, ischemic stroke, and cerebral hemorrhage.

There are two main types of strokes: ischemic stroke and hemorrhagic stroke. Ischemic stroke, also called cerebral infarction (Figure 1A), occurs because of disrupted blood flow to the brain due to a clot or clots in the vessel. Ischemic stroke causes necrosis in the affected area of brain tissues, leading to irreversible neural damage. Unfortunately, cerebral infarction or ischemic stroke is the most frequent CNS complication following viper envenoming [69] due to abnormal activation of thrombocytes, such as platelet aggregation [70]. Among the proteins in the viper venom, Snake C-type lectin-like proteins (snaclecs) are considered to be the cause of ischemic stroke by activating thrombocytes or platelets [71,72]. Snaclecs are mainly expressed in the venoms of vipers and colubrids [73,74]. Regarding the vipers reported in this review, snaclecs were identified in *Bothrops* [75,76], *Daboia* [77], *Crotalus* [78], and *Trimeresurus* [71]. In addition, procoagulant proteases, one type of snake-venom serine proteases (SVSPs), were reported to be involved in affecting blood coagulation [79,80]. Rather than ischemic stroke, hemorrhagins are the toxic components of Viperidae snake venom, which were reported to cause endothelial damage and an increase in vascular permeability, leading to hemorrhagic stroke [81,82]. Here, we review 13 cases of ischemic stroke or cerebral infarction, depending on the authors’ preference, followed by viper envenoming.

Among five patients diagnosed with ischemic stroke after snakebite, four of them were bitten by *D. russelii*. They received AV treatment in the hospital and recovered with no or minor complications [36,37,38]. Although SVMPs and hemotoxins are the main toxins in the venom of *D. russelii*, two patients were also diagnosed with neural disorders such as ptosis, seizure, and speech disturbances [38]. These may have been caused by the neurotoxins in the venom. PLA_2_ is the most abundant component in the venom of *D. russelii.* The proteolytic activity of PLA_2_ promotes degradation and depletion of fibrin (ogen) resulting in a coagulopathy [83].

Another 5-year-old girl in Morocco was bitten by *Cerastes* and sent to the hospital 4 days later. She was diagnosed with ischemic stroke and developed thrombocytopenia and acute anemia [13]. She did not receive any AV treatment and died on day 7. Unavailability of the AV and delayed hospitalization may have been the causes of her fatality.

In India, four patients were bitten by *D. russelii* and diagnosed with cerebral infarction during hospitalization [12,39,40,41]. Three patients received AV treatment; two of them recovered with no complications. Unfortunately, one patient died even after 35 vials of AV administration due to misidentifying the snake species at the beginning of treatment [12]. One patient did not receive any AV and recovered with motor deficit [41]. The neurotoxins of *D. russelii* also caused neural complications such as Gerstmann’s syndrome [40] and ptosis [12] in patients.

In Martinique, in the French Caribbean, a case of *Bothorps Ianceolatus* snakebite occurred in a 74-year-old man who was sent to the hospital where he lost consciousness for 2 days. Specific AV treatment was given without reaction. A magnetic-resonance imaging (MRI) diffusion-weighted scan showed multiple cerebral infarcts, and he died 10 days later [11]. The likely cause of death was cardiogenic shock resulting from massive myocardial infarction. The fatal outcome, in this case, may have been avoided by early antivenom therapy [84].

A case report in Morocco showed that two patients were diagnosed with cerebral infarction after *C. Cerastes* bites [13]. The 32-year-old woman was sent to a local hospital one day after snakebite. She developed generalized tonic-clonic seizures on her way to the hospital. She did not receive any AV treatment and died eventually. Another 51-year-old man was sent to hospital with hemodynamic shock. He did not receive any AV but was treated with fresh frozen plasma and units of platelet administration. Fortunately, he was discharged with full recovery.

A 49-year-old woman in China was bitten by *T. stejnegeri* and sent to the hospital, where she received AV treatment. She had speech disturbances during hospitalization and was diagnosed with cerebral infarction [18]. After 2 weeks in the hospital, she was discharged and followed up 3 months later with a full recovery.

### 6.2. Brain Hemorrhage

SVMPs are zinc-dependent enzymes belonging to the metzincin family [85] that are mainly expressed in *Viperinae* and *Crotalinae* [6]. Based on the size and domain structure, three known SVMPs are identified in P-I (20–30 kDa), P-II (30–60 kDa) and P-III (60–100 kDa) classes [7,75]. In some vipers, SVMPs and anticoagulant toxins exist in their venoms [75]. Once bitten by those vipers, patients are likely to suffer from a severe cerebral hemorrhage (Figure 1B) due to vascular endothelium damage and coagulation disorder.

In Brazil, a 52-year-old man was sent to the hospital 2 days after a juvenile *B. jararaca* bite. He was treated with AV and diagnosed with intracranial hemorrhage because of the proteolytic, hemorrhagic, and coagulant activity in the venom [10]. An earlier case reported that the venom of juvenile *Bothrops* shows higher hemorrhagic but lower proteolytic activity than adult *Bothrops* [86], which explains why this patient had intracranial bleeding without overt local manifestations. Luckily, he was discharged after having recovered. Observing the cases above, only one patient survived following a snakebite from a juvenile *Bothorps*. Another 59-year-old woman who became the victim of a *Bothorps atrox* bite was sent to the hospital one and half hours after a snakebite and was immediately treated with AV. However, she died on the second day due to severe subarachnoid hemorrhage [19].

A 54-year-old man in Florida was bitten by *C. adamanteus* and sent to the hospital within an hour, with immediate AV treatment. He was diagnosed with cerebral edema and hemorrhage [14]. Although he received 16 vials of AV, he still died on day 7. The patient in this case responded very well to AV and was prepared to return home on day 5. However, multiple small cerebral hemorrhages occurred on the morning of day 6; on day 7 he passed away. A 48-year-old woman in India was bitten by an unidentified snake and was diagnosed with intracranial hemorrhage and exotropia in the hospital [20]. She received AV treatment and transfusion of fresh frozen plasma to correct the suspected venom-induced consumptive coagulopathy. Ten days later, she was discharged and recovered with minor visual complications. Based on the hemotoxic and neurotoxic responses, she was likely bitten by *D. russelii*.

### 6.3. Acute Demyelinating Encephalomyelitis (ADEM)

Acute demyelinating encephalomyelitis (ADEM) is an autoimmune-mediated nervous-system disease often occurring after animal or insect bites, which is usually seen in children [87]. However, a 36-year-old man was bitten by *D. russelii* and diagnosed with ADEM on day 8 [42]. He stayed in the hospital for 20 days, receiving 30 vials of AV, and was discharged with full recovery. Adverse reactions to AV such as anaphylaxis (which happens within 10–180 min) and immune complex diseases (happens within 5–25 days) were reported [88]. Due to the delayed observation (day 8) of ADEM in the patient, the authors of this case report postulated that the inflammatory responses due to the AV were a more likely cause of ADEM than direct neurotoxicity of *D. russelii* venom.

In China, a 50-year-old man was bitten by *D. acutus* and sent to the hospital one hour after the snakebite. He experienced severe pain and was treated with AV. Eventually, he was diagnosed with ADEM [21]. After appropriate medical care, he was discharged and followed up one month later with full recovery. Although SVMPs and hemotoxin are present in snake venom, his treatment with AV and other medicines in a very short period minimized venomous toxicity.

### 6.4. Acute Hemorrhagic Leukoencephalitis (AHL)

Similar to ADEM, acute hemorrhagic leukoencephalitis (AHL) is a fulminant inflammatory disease of cerebral white matter. It is more commonly seen in adults and has a high rate of fatality [89]. A 44-year-old man was bitten by *D. russelii* and later diagnosed with AHL [43]. Despite multiple hemorrhagic foci in his brain, he recovered rapidly with AV treatment. He was discharged after 28 days with great improvement.

### 6.5. Posterior Reversible Encephalopathy Syndrome (PRES)

Posterior reversible encephalopathy syndrome (PRES) is a rare syndrome characterized by multiple symptoms such as headaches, altered consciousness, and focal neurological deficits [69]. Many reported cases of PRES are caused by animal bites [90], scorpion stings [91], and wasp attacks [92]. A case in Egypt showed that a 23-year-old pregnant woman was bitten by *C. Cerastes* and sent to the hospital immediately, where she received AV treatment. Among her complications, she complained of a reduction in visual acuity. Based on CT imaging, a very faint hypodense area in the posterior part of the parietal and occipital lobes was found and she was diagnosed with PRES [44]. After one week, she was discharged with significant improvement. Although the author claimed that the underlying mechanism of PRES was unclear in this case, PLA_2_ in the *C. Cerastes*’ venom seems likely to have been the cause of PRES.

### 6.6. Early Morning Neuroparalytic Syndrome (EMNS)

Two rare cases of EMNS were diagnosed in Indian Krait bites. The patients who suffer from EMNS usually do not have bite marks on their bodies, which makes the diagnosis complicated [93]. We also review a case of *Naja* bite, which caused a case of temporary brain death that was rescued by 50 vials of AV [31]. In fact, *Bungarus* and *Naja* bites commonly cause death in India due to neurotoxin-induced respiratory muscle paralysis [94]. On the contrary to viper bites, more than 50% of the elapid bites in this review were on the hand, suggesting that the patients may have tried to capture them.

### 6.7. Other Complications by Snakebite Envenoming

Hemotoxins and SVMPs are the main toxins in the venom of viper snakes [95]. Most vipers generate both SVMPs and hemotoxins. For example, both coagulant enzyme [8,96] and anticoagulant enzyme [97,98] were identified in *Bothrops’* venom. Due to both hemorrhagic and coagulant features of the venoms, viper bites examined in the 21 cases of this review caused cerebral infarction, ischemic stroke, and cerebral hemorrhage, leading to six deaths and five recoveries with complications. Another interesting observation is that most viper bites happened on the foot (17/21), suggesting that most victims were bitten while standing or walking.

In addition to cerebral complications, complications in the spinal cord (a part of CNS) also occurred after snakebite envenoming. Acute-onset flaccid quadriparesis [29] and acute flaccid paraplegia [99], which would cause weakness of the respiratory and pharyngeal muscles, were reported by snakebites of elapid and viper, respectively. The snake PLA_2_ also induces inflammatory responses, leading to spinal pain [100,101]. Another article further confirmed the effects of PLA_2_ on CNS complications [102]. However, a case of spinal-cord complication (myelopathy) after snakebite in India was the result of AV treatment [103]. Thus, the necessity of using AV will be a critical determination for snakebites due to the tiny amount of venom injection.

In addition, other inflammatory responses such as ADEM and AHL are other complications that are noted in viper bites; however, these might be due to the side effects of AV treatment. Among the cases in this review, a rare case of viper bite was diagnosed with PRES, which may have been the result of a minor or undetectable cerebral infarct or hemorrhage.

Ocular complications also occur after hemotoxic envenoming. A review article indicated that subconjunctival hemorrhage, vitreous hemorrhage, central retinal artery occlusion (CRAO), and macular infarction were diagnosed in patients who suffered from viper bites [34]. Rarely, few patients are diagnosed with acute-angle-closure glaucoma (AACG) after viper bites [104,105], which might be due to ciliary-body edema-induced intraocular pressure elevation.

Neurotoxins are the main component of venom in *Elapidae* [32]. In this review, most patients who showed neurotoxic symptoms were bitten by elapids (Table 2). However, neurotoxic influences were also observed in some viper bites such as *D. russelii* [12,38] and *C. cerastes* [13,44] (Table 3). Among the neurotoxins, PLA_2_ and its isomers are identified in the venom of *D. russelii* and *C.* cerastes [106,107]. Fortunately, most neurotoxic influences are recoverable after appropriate treatment. In this review, there is only a rare death caused by *B. multicinctus* bite due to lack of AV at that time [27]. Most of the patients had respiratory paralysis, ptosis, cerebral anoxia, and hypoxic encephalopathy. Not only in viper bites, PRES was also observed in the elapid bite [29], suggesting that PRES is a common syndrome of snakebite, no matter whether it is neurotoxic or hemotoxic. However, not every neurotoxic envenoming causes severe complications. In some cases of snakebites, it merely causes local PNS numbness on the bitten finger [48].

## 7. Treatment

To date, intravenous injection of AV is the most effective treatment for snakebites [108]. In this review, of the six patients who did not receive AV treatment, four of them died. However, some patients were not able to complete the whole AV treatment process due to hypersensitive reactions to AV [28]. Although treated with AV, the misidentification of the snake species at the beginning causes irreversible damage, leading to a fatality [12]. Such iatrogenic causes of snakebite, either by a mistake in diagnosis [12] or by mistreatments [109,110], make the cases worse. Neostigmine, a cholinesterase inhibitor for overcoming paralysis of skeletal muscle, is commonly used to cotreat with AV when patients have neural disorders such as ptosis and muscle weakness [26,29,30,31,38,40]. Hemotoxin-induced ischemic stroke and cerebral infarction seem to be the main CNS complications (15/21) following a viper bite. Aspirin is a known anticoagulant agent and is used against ischemic stroke [111] and cerebral infarction [112]. Thus, aspirin could be an adjuvant for viper bite. SVMPs are the main components in the venom that lead to hemorrhage by proteolytic destruction of the basal membrane and extracellular matrix around the capillary and vessels [113]. Several studies have reported that using chelators to neutralize the cations such as Ca^2+^, Mg^2+^, and Zn^2+^, the cofactors in the active sites of the hemotoxic enzymes, could be a potential therapeutic strategy [114,115,116]. Although EDTA and DTPA can neutralize the hemotoxic activities in vitro, no significant improvement was observed in tissue necrosis or increases of survival in vivo [117]. Moreover, excessive administration of EDTA may cause electrolyte imbalance in patients. A previous study showed that a premix of sodium silicate complex (SSC) with PM could reduce subdermal hemorrhage and elongate the survival in a rodent model of PM envenoming [17], suggesting that SSC may not only inhibit hemotoxic effects but also alleviate neurotoxic influences. Thus, SSC could be an adjuvant as first aid for viper snakebites.

Recently, a promising study showed that 2,3-dimercapto-1-propanesulfonic acid (DMPS), a strong metal chelator, can block the enzymatic activity of SVMPs in vitro and prolong the survival rate of animals that suffered from snake-venom envenoming [118]. Varespladib (LY315920) was demonstrated to show PLA_2_ inhibition against 55 to 60 snake venoms and significantly increase the survival rate of venom-injected rodents [119,120,121]. Later, an orally bioavailable prodrug of Varespladib (methyl-Varespladib; LY333013) was developed and it shows similar efficacy in abrogating or delaying neurotoxic manifestations induced by snake venoms [122]. Both Varespladib [123] and DMPS [124] are in clinical trials for snakebite. In addition, an animal experiment reported that hyperbaric oxygen (HBO) is a potential adjuvant along with AV, showing more prominent protective effects on brain damage caused by *D. acutus* envenoming [125]. α-neurotoxin causes a reversible blockage of acetylcholine receptors, leading to central-nervous-system disorders [126]. A synthetic recombinant peptide against short-chain α-neurotoxin (ScNtx) was developed to neutralize the venoms of diverse genera such as *Micrurus*, *Dendroaspis*, *Naja*, and *Walterinnesia* [127]. Another proteomics study further pointed out the protein landscapes of the venom, which provides a platform for synaptic inhibitor development [128].

## 8. Discussion and Conclusions

**Figure 2:**
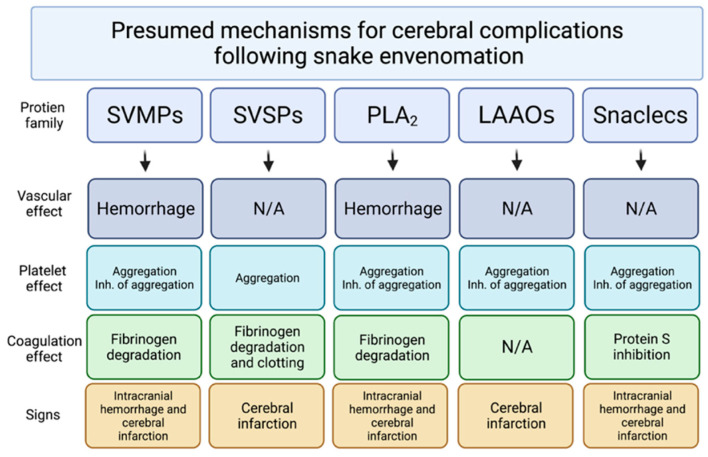
Presumed mechanisms for cerebral complications following snakebite envenoming. **SVMPs**: Snake-venom metalloproteinases; **SVSPs**: Snake-venom serine proteases; **PLA_2_**: Phospholipase A2; **LAAOs**: L-amino-acid oxidase; **Snaclecs**: Snake C-type lectin-like proteins; **N/A**: Not applied. The figure is adapted from the previously described [75].

Cerebral infarctions and hemorrhages are the main causes leading to fatal cases of viper bite. Both hemotoxins and neurotoxins contribute to those severe lethal brain complications (Figure 2) [75]. Although most patients were bitten on the foot or toe by vipers (17 of 21), the toxins still circulate to the brain and cause cerebral complications. To reduce accidental bites, always wearing shoes and pants while walking could be a strategy for preventing viper snakebites. Another rare case of viper (*Protobothrops muscrosquamatus*) snakebite to the head did not show any cerebral complications [129], suggesting that the site of the bite close to the brain may not be associated with snakebite-envenoming-induced cerebral complications. In addition, delayed treatment due to initial misidentification of the snake can cause irreversible tragedy [12]. Identification of features between venomous and nonvenomous [2] snakes might also be helpful to avoid snakebite envenoming and provide the best information for treatment. Finally, in a total of 33 cases of viper and elapid bite, 6 patients did not receive AV, leading to 3 deaths; this indicates that immediate hospitalization and treatment with AV remains crucial for survival after snakebite.

## Figures and Tables

**Table 1:** Brain and nervous-system complications following *Viperidae* envenoming.

Complications	Snake Species	Sex/Age	Bite Mark	Other Symptoms	Presuming Venom Components	Outcome	Antivenom/Amount	Ref.
**Cerebral infarction**	*Bothrops lanceolatus* (Fer-de-Lance)	M/74	Elbow	Atrial fibrillation	**SVMPs**, Hemotoxin	Dead	IV Antivenom/80 mL	[11]
**Cerebral infarction**	*Trimeresurus stejnegeri* (Asian palm pit viper)	F/49	Foot	Speech disturbances	**SVMPs**, Hemotoxin,	Recovered	IV Antivenom/3 vials	[18]
**Intracranial hemorrhage**	*Bothrops jararacussu* (Jararacussu)	M/52	Foot	Sudden loss of consciousness	SVMPs, Hemotoxin	Recovered	IV Antivenom/Unclear	[10]
**Subarachnoid hemorrhage**	*Bothrops atrox* (Fer-de-Lance)	F/59	Foot	Hypothermia, Bradycardia and Hypotension	**SVMPs**, Hemotoxin	Dead	IV Antivenom/80 mL	[19]
**Cerebral edema and hemorrhage**	*Crotalus adamanteus* (Eastern diamondback rattlesnake)	M/54	Hand	Facial fasciculations	**SVMPs**, Hemotoxin, Neurotoxin	Dead	IV Antivenom/16 vials	[14]
**Intracranial hemorrhage**	Unidentified species	F/48	Hand	Exotropia	**SVMPs**, Hemotoxin,	Recover with minor visual complication	IV Antivenom/Unclear	[20]
**ADEM**	*Deinagkistrodon acutus*	M/50	Foot	Severe pain	**SVMPs**, Hemotoxin	Recovered	IV Antivenom/1 vial	[21]

**ADEM**: Acute demyelinating encephalomyelitis; **AHL**: Acute hemorrhagic leukoencephalitis; **SVMPs**: Snake-venom metalloproteinases; **PLA_2_**: Phospholipase A2.

**Table 2:** Brain and nervous-system complications following *Elapidae* envenoming.

Complications	Snake Species	Sex/Age	Bite Mark	Other Symptoms	Presuming Venom Components	Outcome	Antivenom/Amount	Ref.
**Respiratory paralysis, ptosis**	*Bungarus candidus* (Malaya Krait)	M/35	Hand	Tachycardia, prolonged micturition	Neurotoxin, **PLA_2_**	Permanent mydriasis	IV Antivenom/6 vials	[26]
**Respiratory paralysis, ptosis**		M/41	Hand	Tachycardia		Permanent mydriasis	IV Antivenom/3 vials	
**Prolonged cerebral anoxia**		F/12	Hand	Periodic convulsions		Permanent brain damage	Unclear	
**Hyponatremia and cerebral edema**	*Bungarus multicinctus* (Many-banded Krait)	F/17	Foot	Seizure	Neurotoxin	Dead	**N/A**	[27]
**Hypoxic ischemic encephalopathy**	*Bungarus caeruleus* (Indian Krait)	M/15	Foot	Paraplegia	Neurotoxin	Loss of vision	IV Antivenom/1 vial	[28]
**PRES**	*Bungarus caeruleus* (Indian Krait)	M/10	Ear	Hypertension	Neurotoxin	Recovered with vision blurring	IV Antivenom/10 vials	[29]
**EMNS**	*Bungarus caeruleus* (Indian Krait, prediction)	M/38	**N/K**	Difficulty in swallowing and double vision	Neurotoxin	Recovered	IV Antivenom/9 vials	[30]
**EMNS**		F/27	**N/K**	Mydriasis		Recovered	IV Antivenom/Unclear	
**Temporary brain death**	*Naja haje arabica* (Arabian cobra)	F/57	Hand	Breathing ceased	Neurotoxin	Recovered	IV Antivenom/50 vials	[31]

**EMNS**: Early-morning neuroparalytic syndrome; **N/A**: Not Applied; **N/K**: Not known; **PLA_2_**: Phospholipase A2; **PRES**: Posterior reversible encephalopathy syndrome.

**Table 3:** Brain and nervous-system complications following *Viperidae* and *Lamprophiidae* bite: both hemotoxic and neurotoxic venoms.

Complications	Snake Species	Sex/Age	Bite Mark	Other Symptoms	Presuming Venom Components	Outcome	Antivenom/Amount	Ref.
**Ischemic stroke**	*Daboia russelii* (Russell’s Viper)	M/18	Foot	Sever pain	Hemotoxin	Recovered with minor complications	IV Antivenom/6 vials	[36]
**Ischemic stroke**	*Daboia russelii* (Russell’s Viper)	F/40	Foot	Hypotonia of the limbs	**SVMPs**, Hemotoxin	Significant ataxia of gait	IV Antivenom/24 vials	[37]
**Ischemic Stroke**	*Daboia russelii* (Russell’s Viper)	M/70	Foot	Ptosis, Seizure	Hemotoxin, Neurotoxin	Recovered	IV Antivenom/20 vials	[38]
**Ischemic Stroke**		M/55	Foot	Ptosis, Speech disturbances	Hemotoxin, Neurotoxin	Recovered	IV Antivenom/35 vials	
**Ischemic stroke**	*Cerastes cerastes* (Desert horned viper)	F/5	Foot	Thrombocytopenia, Acute anemia	SVMPs, Hemotoxin, **PLA_2_**	Dead	**N/A**	[13]
**Thalamic and cerebral infarctions**	*Daboia russelii* (Russell’s Viper)	M/55	Foot	**N/A**	**SVMPs**, Hemotoxin	Recovered	IV Antivenom/26 vials	[39]
**Cerebral infarction**	*Daboia russelii* (Russell’s Viper)	F/27	Foot	Gerstmann’s syndrome	Hemotoxin, Neurotoxin	Recovered	IV Antivenom/Unclear	[40]
**Cerebral infarction**	*Daboia russelii* (Russell’s Viper, prediction)	M/50	Foot	Status epilepticus	**SVMPs**, Hemotoxin	Motor deficit	**N/K**	[41]
**Ischemic brain infarction**	*Daboia russelii* (Russell’s Viper) Mis-identified at beginning	M/43	Foot	Ptosis and ophthalmoplegia	**SVMPs**, Hemotoxin, Neurotoxin	Dead	IV Antivenom/35 vials	[12]
**Cerebral infarction**	*Cerastes cerastes* (Desert horned viper)	F/32	Hand	Tonic-clonic seizures	**SVMPs**, Hemotoxin, PLA_2_	Dead	**N/A**	[13]
**Cerebral infarction**		M/51	Foot	Hemodynamic shock		Recovered	**N/A**	
**ADEM**	*Daboia russelii* (Russell’s Viper)	M/36	Foot	Hematemesis and gum bleeding	SVMPs, Hemotoxin	Recovered	IV Antivenom/30 vials	[42]
**AHL**	*Daboia russelii* (Russell’s Viper, prediction)	M/44	Foot	Hemorrhagic necrosis	**SVMPs**, Hemotoxin	Recovered	**N/K**	[43]
**PRES**	*Cerastes cerastes* (Desert horned viper)	F/23	Foot	Reduction in visual acuity	SVMPs, Hemotoxin,**PLA_2_**	Recovered with visual impairment	IV Antivenom/Unclear	[44]
**Myokymia**	*Crotalus oreganus abyssus* *(Grand Canyon rattlesnake)*	M/46	Hand	Pain, swelling, and erythema of his left hand	Hemotoxin, Neurotoxin	Recovered	IV Antivenom/20 vials	[45]
**fasciotomy-requiring compartment syndrome**	*Rattlesnake*	W/32	Hand	Somnolent, febrile, suffering of headache, tachypnoea	Hemotoxin, Neurotoxin	Recovered with complications	**N/A**	[46]
**Bilateral ptosis**	Crotalus cerastes (sidewinder rattlesnake)	M/56	Foot	Burning and tingling pain	Hemotoxin, Neurotoxin	Recovered	**N/A**	[47]
**Numbness**	*Lamprophiidae* *Homoroselaps lacteus* (Spotted harlequin snake)	M/Adult	Palm	Slight burning sensation, swelling	**SVMPs**, **PLA_2_**	Recovered	**N/A**	[48]
**Subcutaneous ecchymoses**		M/Adult	Thumb	Swelling		Recovered	**N/A**	

**ADEM**: Acute demyelinating encephalomyelitis; **AHL**: Acute hemorrhagic leukoencephalitis; **SVMPs**: Snake-venom metalloproteinases; **N/A**: Not Applied; **N/K**: Not known; **PLA_2_**: Phospholipase A2; **PRES**: Posterior reversible encephalopathy syndrome.

**Table 4:** Complications following rear-fanged *Colubridae* envenoming.

Complications	Snake Species	Sex/Age	Bite Mark	Other Symptoms	Presuming Venom Components	Outcome	Antivenom	Ref.
**Dizziness**	*Oxybelis fulgidus* (*Colubridae*)	M/67	Arm	Moderate pain, tachycardiac, local bleeding	Hemotoxin, **3FTx **(fulgimotoxin)	Recovered	**N/A**	[57]
**Hand edema**	*Leptodeira annulata* (*Colubridae*) Banded cat-eye snake	F/29	Hand	Burning sensation and itching, pain	**SVMPs**, Hemotoxin	Recovered	**N/A**	[58]
**CRPS**	*Leptodeira annulata* (*Colubridae*) Banded cat-eye snake	M/44	Arm	Fever, chills, nausea, and light-headedness.	SVMPs, Hemotoxin, **PLA_2_**	Recovered	**N/A**	[59]

**CRPS**: Complex regional pain syndrome; **PLA_2_**: Phospholipase A2; **SVMPs**: Snake-venom metalloproteinases; **3FTx**: Three-finger toxins, **N/A**: Not Applied.

## Data Availability

The data presented in this study are available in this article.
